# The efficacy of the current adult observation chart: audit of compliance with trust guidelines at City Hospital, Birmingham, UK

**DOI:** 10.1186/1753-6561-9-S1-A38

**Published:** 2015-01-14

**Authors:** MJ Connelly, Bleasdale

**Affiliations:** 1 The University of Birmingham, Birmingham, UK; 2 Sandwell and West Birmingham Hospitals, NHS Trust, UK

## Background

The Royal College Of Physicians (RCP) has shown that the multiplicity of locally developed Early Warning Scores (EWS) used throughout the UK is causing a ‘lack of consistency in detecting the deterioration of patients [1]. The RCP therefore produced a report in July 2012 proposing a National Early Warning Score (NEWS), a point based system, with the aim of standardisation across the UK. However, there is concern that if locally developed systems are working well why “fix what isn’t broken”. The current system used at Sandwell & West Birmingham Hospitals (SWBH) NHS Trust, UK is a simple colour coded observation chart. As observations become more abnormal they are documented in amber or red zones on the chart. Instructions on the chart explain that single “amber”, double “amber” or “red” observation should trigger a specified action, to be documented in the clinical notes, and increased frequency of observations. Previous audits of patients admitted to the Intensive Care Units of SWBH indicated that even this simple colour coded system was not accurately followed and could indicate that the NEWS, a more complicated point based system, may worsen compliance. The aims of this audit were to evaluate adherence to the current colour-coded EWS adult observation chart across a variety of wards.

## Methods

194 patients charts were reviewed during the 2012 winter period. For each patient, all observations over the preceding 24hrs were assessed. If they indicated that further action should be taken, the nursing and medical notes were consulted.

## Results

25% of patients had an incomplete set of observation parameters recorded. For single “amber” observations only 21% had any subsequent action recorded and 67% had their frequency of observations increased. For double “amber” observations the results were 33% and 83%. Of the 11 red observations, only 3 had actions documented.

## Conclusions

The results highlight that the current chart does not appear to be as effective or utilised in the way that it was designed. To re-establish effective use of the current chart will require a massive trustwide education programme. The proposed RCP NEWS system has been tested and demonstrated to be an effective system for identifying at risk patients. It may make more sense to concentrate education efforts on the introduction of the RCP NEWS system rather than re-launching the old one.
